# Nutraceuticals for Preventing and Managing Cancer Chemotherapy–Induced Cardiotoxicity: An Updated Comprehensive Review

**DOI:** 10.1155/crp/5771319

**Published:** 2026-08-17

**Authors:** Seyedeh-Tarlan Mirzohreh, Samad Ghaffari, Naimeh Mesri Alamdari, Erfan Banisefid, Melika Ghaffari, Arvin Namazi Shabestari, Armin Imanzadeh Ghazijahani, Neda Roshanravan, Faezeh Tarighat

**Affiliations:** ^1^ Cardiovascular Research Center, Tabriz University of Medical Sciences, Tabriz, Iran, tbzmed.ac.ir; ^2^ Faculty of Medicine, Tabriz University of Medical Sciences, Tabriz, Iran, tbzmed.ac.ir; ^3^ Endocrine Research Center, Tabriz University of Medical Sciences, Tabriz, Iran, tbzmed.ac.ir; ^4^ College of Science, University of Tehran, Tehran, Iran, ut.ac.ir

**Keywords:** cardio-oncology, cardioprotection, chemotherapy-induced cardiotoxicity, nutraceuticals

## Abstract

Chemotherapy‐induced cardiotoxicity remains a major barrier to durable cancer control and survivor well‐being, arising from oxidative stress, mitochondrial injury, inflammation, and emerging pyroptotic pathways. This updated narrative review integrates mechanistic and clinical data on nutraceuticals, including amino acids, fatty acids, vitamins, minerals, and polyphenols. Consistent clinical signals include omega‐3 fatty acids, alpha‐lipoic acid, vitamin D, coenzyme Q10, black seed oil, and vitamin E with levocarnitine, which primarily reduce oxidative stress to protect against cardiotoxicity. Additional preclinical and early clinical work highlights vitamin C–, taurine‐, zingerone‐, and rosemary‐derived phenolics as candidates that target oxidative and inflammatory injury without compromising antitumor activity. Translation to care should be prevention‐focused, pairing baseline risk assessment with surveillance that utilizes high‐sensitivity troponin, natriuretic peptides, and advanced echocardiography, such as global longitudinal strain. Nutraceuticals should complement rather than replace established oncologic therapy and cardioprotection, with integration guided by individual risk and ongoing monitoring.

## 1. Introduction

Cancer treatment has improved significantly in recent years, and it has been proven to increase the rate of cure in cancer and raise the number of cancer survivors, as well as decrease recurrence. Chemotherapy and radiotherapy used in cancer treatments are well‐recognized causes of future cardiac complications. However, the applicability of these drugs is limited by the risk of cardiotoxicity, which limits the survival rate and quality of life of cancer survivors [1].

Cardiotoxicity refers to heart damage caused by cancer treatments and is a major limitation to using drugs such as anthracyclines and trastuzumab, which can lead to life‐threatening cardiomyopathy [2]. It is a serious adverse effect of certain chemotherapeutics, increasing morbidity and mortality in cancer survivors [3].

Defined as reduced left ventricular ejection fraction (LVEF), heart failure (HF) symptoms, structural or cellular cardiac injury, conduction or vascular abnormalities, hypertension, and ischemia, cardiotoxicity can occur early or late. Presentations range from asymptomatic biomarker or imaging changes to severe outcomes such as HF, arrhythmias, or sudden cardiac death [4].

Numerous studies have shown that the type of chemotherapeutic drug significantly influences the risk of cardiotoxicity. Proposed mechanisms include direct cellular toxicity with cumulative myocardial injury (MI) causing systolic and diastolic dysfunction, coagulation system effects leading to ischemia, thrombosis, and vascular damage, as well as arrhythmogenesis, hypertension, myocardial/pericardial inflammation, and oxidative stress [5, 6]. A key molecular pathway involves excessive reactive oxygen species (ROS) production [7], causing lipid, DNA, and protein damage [4], mitochondrial injury [8], inflammation [9], and cardiomyocyte apoptosis [4]. Because cardiomyocytes have abundant mitochondria but low superoxide dismutase (SOD) and catalase (CAT) levels, they are particularly susceptible to oxidative injury. Therefore, strategies to reduce chemotherapy‐induced cardiac damage often focus on limiting oxidative stress [9].

Chemotherapy drugs upregulate the expression of the NOD‐like receptor family, pyrin domain containing 3 (NLRP3 inflammasome), NLRP3, apoptosis‐associated speck‐like protein containing a CARD (ASC), and C‐caspase‐1 and elevate expression of the pyroptotic executors (GSDMD and GSDMD‐N) and proinflammatory cytokines (IL‐1β and IL‐18) [10]. Patients receiving systemic drugs with cardiotoxic potential should consider having serial assessments to monitor heart function at baseline, during treatment, and in some cases, post‐treatment [11].

Cardiovascular complications from chemotherapy may be reduced through preventive strategies such as regular monitoring and cardioprotective agents. No specific guidelines exist for treating chemotherapy‐induced cardiotoxicity, making prevention the main approach. Studies have explored drugs and methods to limit myocardial damage, including prophylactic antioxidants that inhibit chemotherapy‐induced free radical formation [12, 13]. However, no effective targeted therapies, either prophylactic or curative, are available. Based on empirical evidence, some natural compounds show protective effects by regulating redox homeostasis and inducing antioxidant enzyme expression [14].

The study aims to provide a concise and up‐to‐date summary of the natural compounds that can prevent and manage chemotherapy‐induced cardiotoxicity for the busy clinician.

## 2. Method

A literature search was conducted using electronic databases, including PubMed, Cochrane Library, Scopus, and Google Scholar. We aimed to summarize the published literature on the nutraceutical supplements in preventing and managing cancer chemotherapy–induced cardiotoxicity. A combination of the terms including “cardiotoxicity,” “chemotherapy,” “nutraceuticals,” “functional foods,” “nutrients,” “vitamins,” “dietary supplements,” “spices,” “prebiotics,” and “cardiovascular disease” was used. Boolean operators (AND, OR) were applied as appropriate to combine terms.

Case reports, editorial letters, gray literature, unpublished reports, and papers for which full texts were not available in English were considered exclusion criteria. The most recent search was conducted on 18 June 2026.

## 3. Nutraceuticals and Mechanisms of Cardioprotection

Although nutraceuticals act via multiple pathways that overlap in various steps, this study discusses them based on their primary target mechanism.

### 3.1. Scavenging ROS

#### 3.1.1. N‐Acetylcysteine (NAC)

NAC is a modified form of the amino acid L‐cysteine that acts as a precursor to glutathione (GSH), a vital antioxidant that protects cells from oxidative damage. By increasing GSH levels, NAC supports various body functions, including immune defense and detoxification. Clinically, NAC is used to treat acetaminophen (paracetamol) overdoses by helping the body neutralize toxic metabolites. Its mucolytic properties also make it effective at loosening thick mucus in respiratory conditions such as chronic obstructive pulmonary disease (COPD) and cystic fibrosis. Beyond these uses, NAC has been studied for its potential benefits in managing psychiatric disorders, supporting liver health, and enhancing fertility, among other health concerns. Khazdooz et al. evaluated NAC in 60 breast cancer patients receiving anthracycline‐based chemotherapy. Patients received either 600 mg/day NAC during treatment or no NAC. One month postchemotherapy, troponin I was detected in 1 NAC patient versus 3 controls (NS), but mean levels were significantly lower with NAC (0.120 ± 0.039 vs. 0.192 ± 0.063; *p* < 0.001), suggesting that NAC may help reduce anthracycline‐induced cardiotoxicity [15]. However, Jo et al. failed to show any significant change in LVEF (*p* = 0.502) and major endpoints (*p* = 0.672) in 103 cancer patients treated with chemotherapy with or without NAC (1200 mg orally every 8 h) in a prospective randomized trial [16]. The absence of significant clinical benefit in this study may be attributed to NAC’s low oral bioavailability and extensive first‐pass metabolism, which limit its systemic and myocardial antioxidant effects.

#### 3.1.2. Betaine

Betaine, a naturally occurring compound derived from glycine, plays a crucial role in several physiological processes, including methylation reactions, osmoregulation, and lipid metabolism. It is commonly found in beets, spinach, and whole grains, but is also produced endogenously. Betaine has gained attention for its antioxidant properties in treating various health conditions, including cardiovascular diseases, liver function, and metabolic disorders [17].

Singh et al. explored the potential of betaine to mitigate doxorubicin (DOX)‐induced cardiomyopathy. Betaine (200 and 400 mg/kg) was coadministered with DOX via oral gavage for 28 days. The results demonstrated that betaine treatment significantly reduced the levels of creatine kinase–myocardial band (CK‐MB), lactate dehydrogenase (LDH), serum glutamic–oxaloacetic transaminase (SGOT), and triglycerides. Betaine also alleviated the increased oxidative stress by restoring SOD, CAT, malondialdehyde (MDA), and nitrite levels. Histopathological analysis confirmed that betaine reversed tissue injury, providing additional support for its cardioprotective properties. On the molecular level, betaine inhibited the DOX‐induced increase in phospho‐p53, phospho‐p38 MAPK, NF‐kB p65, and PINK 1, while enhancing the expression of adenosine monophosphate–activated protein kinase (AMPK) and suppressing Nrf2 expression. Furthermore, qRT‐PCR analysis revealed that betaine treatment reduced the DOX‐induced upregulation of inflammatory genes (TNF‐α, NLRP3, and IL‐6) and fibrosis‐related genes (TGF‐β and Acta2), which contributed to the prevention of cardiac damage. Interestingly, betaine also boosted the mRNA expression of Nrf2, thus promoting the expression of antioxidant proteins and mitigating oxidative damage. This study presents the first evidence that betaine can prevent DOX‐induced cardiomyopathy by inhibiting oxidative stress, inflammation, and fibrosis through the regulation of AMPK/Nrf2/TGF‐β signaling, suggesting that betaine may be considered a valuable therapeutic option for preventing DOX‐induced cardiotoxicity [18]. Current evidence remains limited to preclinical findings, and further investigation is required to determine the translational relevance of these observations in humans.

#### 3.1.3. Carnosine (CAR)

CAR is a naturally occurring dipeptide composed of beta‐alanine and histidine, primarily found in high concentrations in the brain, heart, and skeletal muscles. Its well‐known antioxidant properties make it crucial in protecting cells from oxidative stress and damage. CAR also exhibits antiglycation effects, suggesting its role in preventing age‐related degenerative diseases and metabolic disorders. In addition, carnosine has been shown to modulate various cellular processes, including calcium homeostasis, enzyme activity, and protein synthesis. Due to its multifaceted protective mechanisms, carnosine has gained attention in clinical and therapeutic research, particularly in the context of neurodegenerative diseases, diabetes, and cardiovascular health [19].

Kumral et al. investigated the potential protective effects of CAR and its combination with vitamin E (CAR + Vit E) against DOX‐induced toxicity in the hearts, livers, and kidneys of rats. The rats were treated with CAR (250 mg/kg/day, intraperitoneally) or CAR + Vit E (200 mg/kg of α‐tocopherol, administered intramuscularly every three days) for 12 consecutive days. On the 8th day of treatment, they received a single dose of DOX (30 mg/kg, intraperitoneally). CAR or CAR + Vit E treatment significantly reduced the serum levels of cTnI, ALT, and AST, decreased the pro‐oxidant status, and improved histopathological findings in the tissues. The study concluded that CAR, especially when combined with vitamin E, protects against DOX‐induced toxicity in the heart, liver, and kidneys by improving biomarkers and restoring oxidative balance, as well as alleviating histopathological changes [20]. Although the available evidence supports potential biological activity, further research is needed to establish its use in clinical settings.

#### 3.1.4. Glutamine

Glutamine is the most abundant free amino acid in the human body. It is considered a conditionally essential amino acid during periods of metabolic stress. Glutamine is involved in several metabolic processes: nitrogen transport, nucleotide and protein synthesis, energy metabolism, redox homeostasis, and cellular signaling. It has been studied for its potential cardioprotective and chemotherapeutic effects, particularly in the context of cancer therapy [21]. Recent research has explored its ability to mitigate toxicity associated with treatments such as DOX, suggesting its potential as a therapeutic agent in preventing drug‐induced organ damage [22].

Xue et al. investigated the cardioprotective and chemosensitizing potential of glutamine and ω‐3 polyunsaturated fatty acids (PUFAs) in the context of cancer chemotherapy. The study assessed the antitumor effects and cardiotoxicity of DOX in a MatBIII mammary adenocarcinoma tumor‐bearing rat model, using a cumulative DOX dose of 12 mg/kg. The rats received glutamine (0.35 g/kg) and ω‐3 PUFAs (eicosapentaenoic acid [EPA] 0.19 g/kg and docosahexaenoic acid [DHA] 0.18 g/kg) either alone or in combination, starting 6 days before chemotherapy and continuing until Day 50. The results indicated that glutamine alone effectively mitigated DOX‐induced cardiac damage, lowered serum cardiac troponin I (cTnI) levels, and reduced cardiac lipid peroxidation (LPO) without affecting tumor growth. However, the combination of glutamine and ω‐3 PUFAs did not offer any additional benefit. In fact, the individual advantages of each treatment on cardiotoxicity and chemosensitization were diminished or entirely lost when combined. These findings suggest a complex interaction between glutamine and ω‐3 PUFAs, challenging the assumption that combining beneficial nutrients always produces synergistic effects [22, 23].

#### 3.1.5. Taurine

Taurine is an amino acid containing sulfur. It is found in many mammalian tissues, including the heart, brain, kidneys, and reproductive organs. The concentration of taurine in the heart is about 20 μM, which is much higher than the levels in plasma. Taurine is crucial for many bodily processes. It helps regulate mitochondrial protein production, protect against antioxidants, detoxify, conjugate bile acids, manage fluid balance, stabilize membranes, support cell growth, maintain calcium levels, and signal against cell death. According to a systematic review by Samadi et al. [24], taurine appears to play a protective role against the cardiotoxic effects of chemotherapy by attenuating oxidative stress, inflammation, and apoptosis in cardiac tissue. Coadministration of taurine attenuated cardiotoxicity by enhancing antioxidant defenses, including SOD, CAT, and GSH‐related pathways, while reducing LPO and oxidative stress markers [24, 25]. Histological analyses showed that taurine reduced chemotherapy‐induced heart damage, including degeneration, fragmentation, and bleeding. It also influenced pathways related to cell death and inflammation, as indicated by alterations in caspase activity, Bcl‐2 family proteins, NF‐κB signaling, and proinflammatory cytokines such as TNF‐α, IL‐6, and IL‐1β [24, 26]. Although these findings suggest that taurine may exert cardioprotective effects against chemotherapy‐induced cardiac injury, further research is required to establish its clinical relevance.

#### 3.1.6. Alpha‐Lipoic Acids (ALAs)

ALA is a naturally occurring antioxidant that plays a crucial role in mitochondrial energy metabolism and redox balance. ALA has been extensively studied for its potential therapeutic effects in various oxidative stress‐related conditions, including neurodegenerative diseases, diabetes‐related complications, and cardiovascular disorders [27, 28]. ALA also exhibits anti‐inflammatory effects by modulating key signaling pathways, such as nuclear factor kappa B (NF‐κB) and tumor necrosis factor‐alpha (TNF‐α), which are implicated in cancer‐related toxicity. Given these properties, it has gained attention as a potential adjunct therapy in oncology, particularly for mitigating chemotherapy‐induced adverse effects, such as neuropathy and cardiotoxicity [29].

Werida et al. explored the potential benefits of ALA in reducing paclitaxel‐induced neuropathy and DOX‐related cardiotoxicity in women with breast cancer. This randomized, double‐blind, placebo‐controlled prospective study included 64 patients who were randomly assigned to either a control group (*n* = 32), which received a standard chemotherapy regimen consisting of four cycles of DOX plus cyclophosphamide (administered every 21 days) followed by weekly paclitaxel for 12 weeks along with a placebo or an ALA group (*n* = 32), which received the same chemotherapy protocol supplemented with 600 mg of ALA daily for six months. The study demonstrated that ALA significantly improved neuropathy scores at the 9th and 12th weeks of paclitaxel treatment. Moreover, by the end of chemotherapy, patients in the ALA group showed a significant reduction in biomarkers such as brain natriuretic peptide (BNP), TNF‐α, MDA, and nitrotyrosine (NT). These results indicate that ALA may be a promising adjunct therapy for mitigating paclitaxel‐induced neuropathy and DOX‐associated cardiotoxicity in breast cancer patients [30].

#### 3.1.7. Baicalein

Flavonoids reduce the negative effects of drugs without sacrificing their anticancer action and thereby maintaining their therapeutic efficiency. Baicalein efficiently scavenges ROS produced by DOX [31]. The flavonoids restore cardiac nonenzymatic and enzymatic antioxidants as well as the downregulated intrinsic and NF‐κB–regulated apoptotic pathways linked with cardiomyocyte mortality produced by DOX. Baicalein inverts DOX‐mediated inflammation in the myocardium by blocking NF‐κB signaling [32]. Baicalein preserved cardiac function by maintaining tissue integrity and lowering the elevation of serum biomarkers, including CK‐MB, LDH, aspartate aminotransferase (AST), and alanine aminotransferase (ALT). It lowers oxidative stress, controls inflammation, and may promote cell repair to protect cardiac fibrosis and heart tissue [33]. While these findings are promising, the current evidence is derived primarily from preclinical studies, and their clinical application needs to be investigated.

#### 3.1.8. Rutin

A documented history of heart disease increases the likelihood of cardiotoxicity, a significant limiting factor in anticancer therapy like carfilzomib (CFZ) [34]. With anti‐inflammatory, anticancer, antibacterial, cardioprotective, and antioxidant properties, rutin is the most prevalent bioflavonoid. By preventing platelet aggregation and lowering capillary permeability, rutin thins the blood and enhances circulation [35]. Serum levels of cardiac enzymes (LDH, CK, and CK‐MB), key indicators of cardiotoxicity, were significantly elevated in the CFZ‐treated group compared with the NC group [36]. Rutin therapy, however, mitigated the changes in enzyme activities caused by CFZ. The aforementioned reports concur with earlier research [37]. The downregulation of a‐MHC mRNA expression and the upregulation of b‐MHC and BNP mRNA expressions, which indicate cardiac hypertrophy, provide additional evidence for the CFZ‐induced cardiotoxicity. Myocardial hypertrophy and myocyte disarray are indicated by an imbalance in the expression of hypertrophic genes, including a‐MHC, b‐MHC, and natriuretic peptides [38]. Current evidence regarding rutin is restricted to preclinical studies, and clinical validation is still lacking.

#### 3.1.9. Naringenin (NRG)

NRG is a flavanone primarily found in citrus fruits, such as grapefruit, tomatoes, and cherries. It has a well‐documented profile including antioxidant, anti‐inflammatory, antiapoptotic, hepatoprotective, and cardioprotective activities. Preclinical evidence has demonstrated that NRG alleviates organ toxicity induced by multiple anticancer agents, including cisplatin, cyclophosphamide, paclitaxel, DOX, and 5‐fluorouracil (5‐FU) through the modulation of oxidative stress, inflammatory signaling, and programmed cell death pathways [39, 40].

NRG neutralizes free radicals, binds redox‐active iron, and boosts the Nrf2/ARE transcriptional pathway. This helps restore the activity of protective enzymes in the body. It also targets the inflammatory response that increases cardiotoxicity, which is linked to NF‐κB activation and the overproduction of cytokines (TNF‐α, IL‐1β, IL‐6, iNOS). This effect has been observed across models of heart, liver, kidney, and nerve injuries [41]. Studies by Subburaman et al. in a chronic DOX model (3 mg/kg IV for 10 weeks) confirmed that coadministration of NRG (50 mg/kg/day oral) normalized cardiac biomarkers (LDH, troponin T, MDA), restored antioxidant enzyme activities, and attenuated TGF‐β1, TNF‐α, and IL‐6 mRNA expression, with corroborating histopathological improvement [42, 43]. Notably, Beshel et al. found that NRG reversed DOX‐induced ECG changes, including ST‐elevation, T‐wave changes, and QRS widening, while normalizing hypertensive markers (NO, ACE)[44]. Importantly, in tumor‐bearing models [45], NRG combined with 5‐FU reduced cardiotoxicity while enhancing antitumor efficacy through the AMPK‐mediated regulation of mitochondrial function. This suggests that NRG does not compromise and may even enhance anticancer activity [46]. Although promising, current evidences do not include the application of NRG in the clinical setting.

#### 3.1.10. Anthocyanins

In H9c2 cardiomyocytes, anthocyanidins demonstrated a protective effect against DOX‐induced cytotoxicity. Additionally, none of the anthocyanidins or anthocyanins affected DOX’s chemotherapeutic effects. Compared to their comparable glycosides, anthocyanin aglycons showed greater protection against LPO and DOX‐induced cytotoxicity. The significance of (1) the quantity and location of hydroxyl and methoxyl groups and (2) the presence of sugar moieties on the flavylium skeleton to demonstrate an antioxidant effect has been highlighted in several studies [47]. The compounds cyanidin and delphinidin, which exhibited the strongest defenses against cytotoxicity and LPO brought on by DOX, share an *ortho*‐dihydroxyl moiety (30, 40‐OH) on the flavylium skeleton and penta‐ and hexa‐hydroxyl groups, respectively. Petunidin, on the other hand, which has one methoxyl group and the same hydroxyl groups as cyanidin, had no protective action, indicating that the methoxyl group is harmful to activity. Similarly, one of the key characteristics that determines anthocyanins’ protective efficacy is their glycosylation. Seven glycosides have shown no protective action against toxicity caused by DOX. These findings are consistent with recent observations showing that anthocyanidins’ ability to scavenge radicals is often diminished by glycosylation and methylation [48]. Clinical investigation of anthocyanins as cardioprotective adjuncts during chemotherapy remains absent, and challenges, including pH‐dependent instability of the flavylium cation, rapid intestinal metabolism, and low systemic bioavailability, must be addressed to enable translation into oncological practice [49].

#### 3.1.11. Silymarin

Silymarin may offer protection against cardiotoxicity from the chemotherapeutic drugs, according to clinical trials and in vitro and in vivo investigations [50]. Due to its anti‐LPO properties, silymarin stabilizes heart membranes and prevents the leakage of cardiac enzymes, as indicated by the majority of in vivo studies. Silymarin can counteract GPx and SOD discharge [51]. The regeneration of the antioxidant system relies on increased ATP levels in cardiomyocytes. Silymarin possesses antioxidant properties as it helps preserve ATP. Research has shown that silymarin can effectively inhibit the activity of CK, an enzyme that is released into the bloodstream following cardiotoxicity and serves as a marker of cardiac damage [52]. The Nrf‐2 is motivated by oxidative stress in DOX therapy. Silymarin can stop the production of genes that are dependent on the antioxidant response element (ARE) by binding to the transcription factor Nrf2. Furthermore, silymarin has antioxidant properties and activates the phosphoinositide 3‐kinase and Akt (PI3K/Akt) pathway. Downregulation of c‐myc blocks the translocation of Bax via upregulating sirtuin‐1 and causes cardiac myocytes to produce Bcl‐2. Silymarin contributes to the apoptotic cascade and ultimately shields myocardial cells from DOX‐induced apoptosis. The primary mechanisms of DOX‐induced hepatotoxicity include inflammation, oxidative stress, and apoptosis. By upregulating apoptosis proteins like Bcl‐xL and downregulating the caspase‐3 pathway, preserving antioxidant enzyme levels, lowering proinflammatory mediators like TNF‐α and IL‐6, and controlling liver AMPK signaling, silymarin modulates this DOX complication. AMPK is a type of cellular pathway that may modulate apoptosis, oxidative stress, and inflammation by suppressing the generation of inflammatory cytokines and inhibiting the activation of NF‐κB. Silymarin protects hepatocytes by activating the AKT and AMPK pathways [53].

#### 3.1.12. Piceatannol

Thanks to pleiotropic biological actions, polyphenolic substances have lately drawn the interest of scientists. Piceatannol (*trans*‐3,3′,4,5‐tetrahydroxystilbene) is a naturally occurring polyphenol found in grapes, berries, passion fruit, and wine. It is a structural analog of resveratrol (*trans*‐3,4,5‐trihydroxystilbene). Research has demonstrated that piceatannol possesses a wide range of biological properties, including antioxidant, antimicrobial, anti‐inflammatory, antidiabetic, estrogenic, neuroprotective, and cardioprotective activities [54, 55]. Many human cancer cell lines, including leukemia, lymphoma, melanoma, breast, prostate, bladder, and colon cancer [56, 57], have reported piceatannol to limit proliferation and induce death. Furthermore, shown to trigger autophagy in several cancer cell lines, including MOLT‐4 human leukemia cells and U2OS human osteosarcoma, piceatannol [58, 59]. It has recently been shown to be able to overcome multidrug resistance in numerous cancer cell lines, including adriamycin (ADR)‐resistant HL‐60/ADR cell line. Resveratrol is structurally similar to piceatannol [58, 60, 61]. Although promising in cell line studies, there is currently a lack of clinical evidence regarding the application of piceatannol.

#### 3.1.13. Pterostilbene

Pterostilbene, found in blueberries and grapes, is a naturally occurring demethylated counterpart of resveratrol [62]. Similar to resveratrol, pterostilbene protects the heart and is a strong antioxidant. According to earlier research, pterostilbene may protect the heart from ischemia reperfusion damage, MI, and hypertrophy by inhibiting oxidative stress [63]. Administration of pterostilbene significantly decreased DOX‐induced damage in H9c2 cells in the present study. Furthermore, pterostilbene significantly reduced the immediate cardiac tissue damage that mice experienced from DOX exposure. In both in vitro and in vivo studies, pterostilbene cotreatment significantly reduced DOX‐induced mitochondrial swelling with cristae disorientation and breaking. Furthermore, the administration of pterostilbene reversed the ROS generation and ATP content decrease in H9c2 cells caused by DOX. Cardiomyocytes have a lot of mitochondria to maintain ATP production and heart contractile activity [64]. By increasing ROS production and decreasing SOD and GPx activity in mouse heart tissues, a 6‐day acute DOX treatment dramatically impairs mitochondrial function and causes oxidative stress. Nevertheless, pterostilbene treatment clearly corrected the negative effects above caused by DOX, indicating pterostilbene’s antioxidant and mitochondrial protective activities on cardiomyocytes [65]. Despite encouraging preclinical findings, pterostilbene has not been evaluated in clinical settings.

#### 3.1.14. Genistein

According to recent research, genistein activates the Nrf‐2/HO‐1 pathway, a crucial regulator of the cellular antioxidant defense mechanism, to produce cardioprotective effects. Genistein increases the expression of important antioxidant enzymes such as heme oxygenase‐1 (HO‐1) and NAD (P) H quinone dehydrogenase 1 (NQO1) by upregulating Nrf‐2. Furthermore, genistein mitigates oxidative damage in cardiomyocytes by lowering LPO and ROS levels. In addition, genistein reduces inflammatory mediators such as TNF‐α, IL‐6, and IL‐8, blocks caspase‐3 activation and re‐establishes the equilibrium between proapoptotic and antiapoptotic proteins. It preserves membrane potential and prevents the disturbance of energy metabolism, a defining feature of DOX‐induced cardiotoxicity. Genistein protects mitochondrial function. Coadministration of genistein in mice given DOX significantly lowered cardiac troponin levels. Genistein therapy reduces LPO and ROS production while increasing the expression of antioxidant enzymes. Genistein elevated levels of Nrf‐2, HO‐1, and NQO1 proteins in mice, as well as levels of antioxidant gene expressions [66]. To date, investigations of genistein have been largely limited to experimental studies, with no clinical applications reported.

#### 3.1.15. Curcumin

One dose‐limiting factor influencing the clinical outcome of chemotherapy drugs like DOX is cardiotoxicity. There is increasing evidence that oxidative stress, mitochondrial damage, altered calcium flow, activation of proapoptotic signaling cascades, and other factors contribute to chemotherapy‐induced cardiotoxicity [67]. According to Swamy AV, the repeated use of DOX (15 mg/kg) over a two‐week period raises the levels of cardiac toxicity markers such as LDH and CK. Additionally, it reduces the activity of antioxidant enzymes, including glutathione peroxidase (GPx), CAT, and SOD. Curcumin (200 mg/kg, orally), administered as a pretreatment for two weeks followed by two weeks of DOX treatment, significantly reduced elevated levels of cardiac toxicity indicators [68]. Benzer et al. [69] observed reduced SOD, CAT, and GTP activity, as well as increased CK and LDH, in DOX‐treated rats. For seven days, oral treatment of 100 or 200 mg/kg body weight of curcumin might dramatically reduce serum CK and LDH while increasing SOD, CAT, and GTP activities. Curcumin (30 μM) pretreatment enhanced normal cells’ antioxidant capacity and reduced DOX‐associated cardiotoxicity, according to Sheu et al. [70]. According to Venkatesan, curcumin (200 mg/kg) could prevent the rise in serum CK and LDH caused by ADR and considerably reduce the early signs of ADR‐induced cardiotoxicity, such as an increase in the heart rate and ST segment elevation [71]. According to Bahadır et al. [72], cisplatin resulted in a large rise in MDA levels, a substantial reduction in CAT and SOD activity, and severe cardiac degenerative changes. However, curcumin pretreatment significantly reduced the cardiotoxic symptoms caused by cisplatin [72].

#### 3.1.16. Caffeic Acid

Caffeic acid (3,4‐dihydroxycinnamic acid) is a hydroxycinnamic acid polyphenol found abundantly in coffee, fruits, vegetables, honey, and propolis. The name can be confusing, but it bears no chemical or pharmacological relationship to caffeine. Belonging to the phenylpropanoid family, its biological activity traces back to two structural features: an *ortho*‐dihydroxyphenyl catechol group and a conjugated acrylic acid side chain. These give it a strong capacity to scavenge free radicals, chelate iron, and bind directly to protein targets [73]. Most antioxidants work by intercepting ROS after they have already been generated. Caffeic acid takes a different approach. It binds directly to the Keap1 protein at residues N532 and M550, physically freeing Nrf2 from the repressor complex that keeps it inactive under normal conditions. From there, it pushes Keap1 toward degradation through a p62‐dependent autophagic mechanism, allowing Nrf2 to activate the ARE pathway and drive transcription of HO‐1, NQO1, and the enzymes responsible for GSH synthesis [74]. The result is antioxidant defense built at the source rather than patched together downstream.

Alongside this, caffeic acid suppresses the NF‐κB inflammatory cascade, bringing down TNF‐α, IL‐1β, and hs‐CRP levels. Notably, IL‐10, an anti‐inflammatory mediator, rises at the same time rather than falling with the rest, pointing to active inflammatory rebalancing rather than simple suppression. These changes have been observed both at the cytokine protein level and directly within cardiac tissue.

Mohammad et al. used a rat model of DOX‐induced cardiotoxicity, administering DOX intraperitoneally at 3 mg/kg every other day across 2 weeks. Animals receiving caffeic acid at 40 mg per day showed meaningful recovery: Left ventricular function returned toward normal, plasma troponin I and MDA levels dropped, and cardiac tissue appeared histologically intact with no residual fibrosis [75].

The more complicated picture emerges when caffeic acid is combined with chemotherapy. Sirota et al. found that the timing of administration relative to cisplatin is not a minor detail but rather a determining factor in whether caffeic acid supports or undermines treatment. Given at the same time as cisplatin, it enhances tumor cell cytotoxicity by inhibiting GST and GSR. Given beforehand, it activates Nrf2‐driven resistance in cancer cells and may reduce chemotherapy effectiveness [76]. The same pathway that protects the heart can, under different timing conditions, extend protection to the very cells the treatment is trying to eliminate.

This scheduling sensitivity makes it clear that caffeic acid cannot simply be added to a chemotherapy regimen without forethought. Rigorous pharmacokinetic studies and properly controlled clinical trials are necessary before it can be used safely alongside existing treatment protocols [77].

#### 3.1.17. Propolis

Propolis is a resinous substance produced by honey bees, derived from the leaves, buds, and sap of trees, as well as flower blossoms. The primary components of propolis include flavonoids, organic acids, phenols, various enzymes, vitamins, and minerals [78]. The broad spectrum of biological properties of propolis has led to increased interest in its use as a safe therapeutic agent. Propolis and its active compounds, particularly flavonoids, exhibit antibacterial, analgesic, anti‐inflammatory, antioxidant, immune‐enhancing, and antiproliferative effects in cultured human tumor cells, as well as antitumor activity in murine models. The antioxidant activity of flavonoids is attributed to their ability to directly scavenge free radicals or stabilize ROS through interactions with their reactive components [79]. Flavonoids enhance the activity of endogenous antioxidant enzyme systems, including SOD, CAT, GPx, and GR. Additionally, immune activity enhanced by propolis and its compounds promotes haemopoietic regeneration and survival after radiation‐induced lympho‐ and myelosuppression. The protective effect of propolis on the bone marrow and lymphoid tissue of mice subjected to cytotoxic drugs [80]. The mechanisms underlying radiosensitization induced by RNR inhibitors are presumed to involve cell cycle synchronization and/or the inhibition of DNA repair processes. Propolis supplementation leads to an accumulation of cells in the G1 phase and preferentially induces cell death in the S phase. Radiosensitization may be observed, as cells in the S phase exhibit greater radioresistance compared to those in the G1 phase. The findings of this study indicate that propolis may have a positive impact on cancer radiotherapy [81]. Current research on propolis remains at the preclinical stage, and clinical validation is still lacking.

#### 3.1.18. Quercetin

Quercetin is present in fruits, vegetables, green tea, and red wine. It carries well‐documented antioxidant, anti‐inflammatory, antiapoptotic, and antitumor properties across a broad range of experimental models. It neutralizes ROS without compromising antitumor activity. The DOX‐cyclophosphamide (AC) regimen used in triple‐negative breast cancer (TNBC) generates cardiac oxidative stress through semiquinone radical production and GSH depletion, and quercetin attenuates this through ROS inhibition and ERK1/2 pathway activation in cardiomyocytes. That this same ERK1/2 activation simultaneously enhances AC antitumor activity in TNBC cells points toward a dual‐compartment benefit rather than a simple trade‐off [82]. Cardiac protection at the molecular level operates partly through Bmi‐1, a mitochondrial protective protein whose upregulation coincides with suppression of Bid, p53, and NADPH oxidase. Bmi‐1 silencing abolished these effects entirely, establishing it as the central mediator [83]. A separate mechanism has since been identified: Quercetin blocks ferroptosis in H9c2 cardiomyoblasts with efficacy comparable to ferrostatin‐1, a selective ferroptosis inhibitor, extending its cardioprotective profile into territory not previously associated with flavonoid activity [84]. Manni et al., working in the 4T1 TNBC mouse model, demonstrated that quercetin also potentiates cyclophosphamide antitumor activity through gut microbiome remodeling and an immune shift favoring T cell and NK cell activation alongside reduced regulatory T cell populations [85]. Clinical application remains a future goal. Controlled trials in anthracycline‐treated patients are absent, and quercetin’s poor oral bioavailability presents a persistent barrier to therapeutic use. Chitosan and solid lipid nanoparticle (NP) formulations are under investigation as delivery strategies, although clinical validation of these approaches has not yet been established [86].

#### 3.1.19. Vitamin C

Vitamin C (ascorbic acid) is a water‐soluble antioxidant essential for various physiological functions, including collagen synthesis, immune system regulation, redox homeostasis, and neurotransmitter synthesis [87]. As a potent scavenger of ROS and reactive nitrogen species (RNS), vitamin C plays a critical role in protecting cells from oxidative damage.

Akolkar et al. investigated the cardioprotective effects of prophylactic vitamin C treatment against DOX‐induced cardiac dysfunction, apoptosis, inflammation, and oxidative/nitrosative stress, as well as its impact on vitamin C transporter proteins. Using a rat model, the researchers found that DOX administration (cumulative dose: 15 mg/kg) significantly impaired both systolic and diastolic cardiac function and caused structural myocardial damage. These pathological changes were linked to increased myocardial ROS, reduced antioxidant enzyme activity, upregulation of proapoptotic markers (Bax, Bnip‐3, Bak, and caspase‐3), and heightened inflammatory responses. DOX also aggravated oxidative/nitrosative stress by increasing levels of superoxide, protein carbonylation, LPO, nitric oxide (NO), NO synthase (NOS) activity, protein nitrosylation, and inducible NOS protein expression. Additionally, DOX treatment elevated proinflammatory cytokines, including TNF‐α, IL‐1β, and IL‐6, while downregulating key vitamin C transport proteins, such as sodium‐ascorbate cotransporter 2 and glucose transporter 4. Notably, vitamin C supplementation, both prophylactically and concurrently with DOX, effectively mitigated these harmful effects, improving survival rates, preserving cardiac function, and preventing structural myocardial damage. These findings highlight vitamin C’s potential as a cardioprotective agent against DOX‐induced cardiomyopathy by reducing oxidative/nitrosative stress, inflammation, and apoptosis while maintaining vitamin C transporter protein expression [88].

#### 3.1.20. Vitamin E

Vitamin E is a fat‐soluble antioxidant that plays a crucial role in protecting cells from oxidative damage caused by ROS. As an essential nutrient, vitamin E contributes to immune function, skin health, and the prevention of chronic diseases, including cardiovascular diseases, cancer, and neurodegenerative conditions. Its antioxidant properties are particularly important in protecting cell membranes from LPO, thereby maintaining the integrity of cellular structures and preventing cellular damage [89].

Moustafa et al. conducted a prospective, randomized controlled trial to assess the cardioprotective potential of vitamin E and levocarnitine (EL) in preventing acute DOX‐induced cardiotoxicity in female breast cancer patients. The study included 74 patients receiving DOX and cyclophosphamide (AC), who were randomly assigned to either an EL plus AC group or an AC‐only group for four chemotherapy cycles. The findings revealed that patients in the EL‐treated group had significantly lower levels of B‐type natriuretic peptide and CK compared to the control group. Moreover, the addition of EL led to a 24.2% reduction in cardiac events (*p* = 0.02). The reported adverse effects were mild and manageable. The study concluded that EL appears to be a well‐tolerated and effective prophylactic strategy for reducing acute DOX‐induced cardiotoxicity and emphasized the need for further research, particularly in patients receiving higher cumulative doses of DOX (240 mg/m^2^) [90].

#### 3.1.21. Selenium

Selenium, an essential trace element, plays a crucial role in antioxidant defense, immune function, cellular health, and thyroid hormone metabolism [91, 92]. It is a component of selenoproteins, a group of proteins protecting cells from oxidative damage by neutralizing free radicals. Selenium is obtained through nuts, seafood, and grains. Selenium has been studied for its potential therapeutic effects in cancer, cardiovascular diseases, and inflammation. In recent years, selenium has gained attention for its protective role in mitigating drug‐induced toxicity, particularly in chemotherapy, where it may help reduce oxidative stress and inflammation associated with treatments like DOX [93].

Nadheer Albakaa et al. conducted a study to explore whether selenium could protect against DOX‐induced cardiotoxicity in rats. The study involved 32 male rats, randomly assigned to four groups with eight rats in each. The control group received distilled water, while the DOX group was administered 2.5 mg/kg of DOX three times a week for 2 weeks. Another group was treated with selenium alone at a dose of 1 mg/kg for 2 weeks. DOX caused significant cardiotoxicity, as indicated by substantial increases in cTnI, intercellular adhesion molecule‐1 (ICAM‐1), and caspase‐3 levels. Additionally, levels of GSH and SOD in cardiac tissues were significantly lower compared to the control group. Histological analysis showed cardiac lesions and damage in the DOX‐treated group. Selenium supplementation significantly alleviated these effects, leading to reductions in cTnI, ICAM‐1, and caspase‐3 levels, along with increases in SOD and GSH. Moreover, selenium improved histopathological scores of cardiomyopathies. These findings suggest that selenium effectively mitigates DOX‐induced cardiotoxicity, likely through its actions on oxidative stress, inflammation, and apoptosis [94].

#### 3.1.22. Cyanidin

Cyanidins are a subclass of anthocyanins, a group of water‐soluble flavonoids responsible for the red, blue, and purple pigmentation in various fruits and vegetables, such as berries, grapes, and purple corn. These natural compounds exhibit strong antioxidant properties and have been linked to a variety of health benefits, including anti‐inflammatory, anticancer, and cardioprotective effects. Studies suggest that cyanidin 3‐glucoside (C3G), a major cyanidin derivative, plays a crucial role in mitigating oxidative stress and inflammation, thereby reducing the risk of chronic diseases, including cardiovascular disorders and metabolic syndromes [95, 96].

Petron et al. explored the cardioprotective potential of C3G against DOX‐induced cardiotoxicity in mice. In vitro studies on murine HL‐1 cardiomyocytes revealed that pretreatment with both pure C3G and purple corn extract improved cell survival following DOX exposure. However, neither compound affected DOX’s cytotoxic effects on human cancer cell lines. To further investigate the protective effects of a C3G‐enriched diet in vivo, the study compared two groups of mice: one consuming a diet rich in anthocyanins from purple corn (red diet, RD) and the other fed a diet without anthocyanins (yellow diet, YD). The results showed that mice on the RD had a higher survival rate than those on the YD after receiving a toxic dose of DOX. Additionally, ultrastructural analysis of heart tissue from RD‐fed mice exhibited reduced histopathological damage. These findings indicate that dietary C3G derived from purple corn plays a protective role against DOX‐induced cardiotoxicity in mice [97]. Further clinical studies are required before cyanidin can be considered for therapeutic application.

#### 3.1.23. Rosmarinic Acid (RA) (From Rosemary)

RA has been shown to possess antioxidant, anti‐inflammatory, and anticancer effects. Rahbardar et al. [98] either in vivo (male rats) or in vitro (MCF7 breast cancer cells) studied the cardioprotection against DOX toxicity by RA. The researchers evaluated the cardiotoxic and anticancer effects of the compounds to clarify their role in cardiotoxicity and anticancer activity.

The results indicated that DOX elevated the heart‐to‐body weight ratio, RRI, QA, STI, QRS duration, and voltage. It also decreased the heart rate, blood pressure, maximum and minimum dP/dt, and left ventricular developed pressure (LVDP). Furthermore, DOX raised MDA level, decreased GSH level, and induced fibrosis and necrosis in cardiac tissue. The treatment with RA on the other hand reduced the toxic effect of DOX [98] The translational potential of RA appears promising, but no clinical investigations have yet been reported.

#### 3.1.24. Diosmin

Diosmin (3′,5,7‐trihydroxy‐4′‐methoxyflavone‐7‐rutinoside) is a flavonoid glycoside found principally in citrus pericarp, notable among phytochemicals for its existing clinical approval in Europe as a venoactive agent for chronic venous insufficiency, giving it a translational advantage over most experimental cardioprotectants. Following intestinal hydrolysis, diosmin forms its bioactive aglycone diosmetin, which retains most of diosmin’s biological activities. Diosmin preserves Na/K‐ATPase, an enzyme that DOX selectively damages through oxidative stress. It also suppresses NF‐κB, restores the Bcl‐2/Bax apoptotic balance, and inhibits MMP‐9, a key fibrotic mediator [99]. In rat models, diosmin pretreatment reversed DOX‐induced ECG abnormalities, normalized HIF‐1α and the inflammatory markers IL‐1β and IL‐10, and restored SOD activity while reducing MDA [100]. Its cardioprotective scope is not limited to anthracyclines. The micronized purified flavonoid fraction (MPFF), which is a clinically used diosmin‐hesperidin combination, attenuated cisplatin‐induced cardiac injury through NLRP3 inflammasome suppression and downstream caspase‐1/ caspase‐3 inhibition, while also reversing cisplatin‐associated dyslipidemia and insulin resistance in male rats [101]. Preclinical evidence confirms that diosmin does not compromise DOX’s cytotoxicity against MCF‐7 breast cancer cells, while diosmetin independently suppresses tumor growth across multiple cancer types via FAK and PI3K/Akt/mTOR inhibition, supporting its value as a cardioprotective adjunct without sacrificing antitumor efficacy [100, 102, 103].

#### 3.1.25. Resveratrol (RSV)

According to a recent study, RSV‐induced protection against DOX cardiotoxicity is mediated by autophagy suppression [104]. In the previous investigation, a thorough examination of the involvement of RSV in DOX‐induced cardiotoxicity was conducted, concentrating on the signaling pathways involved, in order to look into this further. It is demonstrated that DOX promoted the production of proapoptotic proteins through p38MAPK/p53 signaling, which in turn prevented AMPK activation and accelerated myocardial cell death. On the other hand, it is demonstrated that RSV phosphorylated/activated ULK1 and suppressed mTOR by activating AMPK in a dose‐ and time‐dependent manner. Autophagy is directly promoted by AMPK/mTOR/Ulk1 pathway activation [105], which may have reduced DOX‐induced cardiotoxicity by destroying toxic substances and damaged cellular organelles. As previously shown in other cell types [106], it has been demonstrated that RSV‐induced autophagy in H9C2 cardiomyocytes was mediated by the suppression of mTOR activity. Additionally, both in vitro and in vivo, combined RSV and DOX treatment reduced DOX‐induced cardiac apoptosis. RSV and autophagy induction are acting defensively in this situation, shielding the heart tissue from DOX’s damaging effects. These results, however, go counter to those of Xu et al. [104], who suggested that one significant mechanism of RSV‐mediated cardioprotection is the inhibition of DOX‐induced autophagy. It is commonly known that, depending on the situation, autophagy activation can either be advantageous or harmful to the heart. The disparities in the results could be caused by variations in the cell lines, animal models, and other elements used in these two investigations. The results of Xu et al. [104] may indicate that there is another mechanism for DOX‐induced autophagy that does not need pAMPK, even though AMPK activation is commonly utilized as a marker of autophagy induction. In light of the other possible pharmacological effects of RSV, it is also necessary to take into account its other possible targets [107]. The principal downstream target of RSV is reportedly Sirt1, which is also referred to as an activator of sirtuins (SIRTs, a type of NAD+‐dependent deacetylase [108]. There might be further unidentified RSV targets, though. While RSV has shown efficacy in experimental models, its applicability in human clinical settings remains unclear.

#### 3.1.26. Black Seed Oil

Black seed oil (*Nigella sativa L*.) is made of unsaturated fatty acids (linoleic acid, oleic acid, and palmitic acid) and thymoquinone (TQ) [109]. Several preclinical studies demonstrated its antioxidant, anti‐inflammatory, and cardioprotective properties [110–113].

Hagag et al. conducted a clinical trial on 40 children with acute lymphoblastic leukemia (ALL). Half of the patients received black seed oil 80 mg/kg/dose starting simultaneously with DOX infusion, while the other group received placebo with DOX infusion. A considerable decrease in systolic function (EF, FS, s wave) was observed in the placebo group. The study implies cardioprotective effects of Black seed oil [114]. These effects can be a result of both TQ and PUFA ingredients [115].

Chen et al. suggested that alleviating oxidative stress and activating the Nrf2/HO‐1 signaling pathway by TQ can decrease ferroptosis (cell death from iron accumulation) and preserve murine cardiomyocytes in the case of DOX‐induced cardiotoxicity [116]. Preliminary human studies have demonstrated potential therapeutic effects of black seed oil; nevertheless, stronger evidence from adequately powered randomized controlled trials is necessary.

#### 3.1.27. Luteolin

Despite extensive investigation into therapeutic mechanisms, the poor solubility of flavonoids has long posed a challenge for scientists. By suppressing viability, migration, angiogenesis, and invasion [117], luteolin is well known to prevent carcinogenesis across a wide spectrum of malignancies. For better use, nevertheless, the combination of protein and flavonoids can help to solve issues with low solubility and stability. Combining luteolin with a soy protein isolate (SPI)–luteolin nanodelivery system, one of the recent investigations chose a SPI prepared by ultrasonic treatment as the embedding wall material. This SPI delivery technique enhanced the use of fat‐soluble active compounds [118] and the luteolin release rate. The results show the synergy effect of luteolin in combination with conventional chemotherapeutics, including 5‐FU and cisplatin. Based on these results, the use of this natural flavonoid with anticancer drugs in future treatment regimens could result in lower doses of the chemotherapeutics and hence fewer side effects. Furthermore, luteolin may even offer some preventive action against chemotherapeutic‐induced toxicities, such as cisplatin‐induced nephrotoxicity [119] or DOX‐induced cardiotoxicity [120].

#### 3.1.28. Capsaicin

Capsaicin is the active compound found in chili peppers, known for its pungent flavor and potential therapeutic properties. It is a naturally occurring alkaloid that interacts with the body’s sensory neurons through the activation of the transient receptor potential vanilloid 1 (TRPV1) channel. Capsaicin has been widely studied for its analgesic, anti‐inflammatory, antioxidant, and anticancer effects. Beyond its traditional use in culinary practices, capsaicin has gained attention for its ability to modulate various physiological processes, such as pain perception, metabolism, and cardiovascular health. Due to its diverse biological activities, capsaicin has been explored as a potential therapeutic agent in treating conditions such as chronic pain, obesity, heart disease, and cancer [121].

Ahmed et al. examined the mechanisms through which capsaicin exerts cardioprotective effects against cyclophosphamide‐induced cardiotoxicity. The study involved multiple groups of rats: a control group that received only the vehicle for 6 days, a positive control group given cyclophosphamide (200 mg/kg) on Day 4, a group that received capsaicin (10 mg/kg) orally for 6 days followed by cyclophosphamide, a group that received a higher dose of capsaicin (20 mg/kg) for 6 days followed by cyclophosphamide, and a group treated with capsaicin alone (20 mg/kg) for 6 days. Both doses of capsaicin significantly reversed cyclophosphamide effects, notably decreasing lactate dehydrogenase and troponin‐I levels. Capsaicin treatment also lowered levels of inflammatory cytokines (IL‐6, TNF‐α), caspase‐3, LPO, and triglycerides, while enhancing antioxidant enzyme activity. The capsaicin‐treated rats exhibited a restoration of oxidative balance and significant protection against cyclophosphamide‐induced cardiomyocyte damage. These findings suggest that capsaicin may offer a protective role in counteracting the harmful cardiovascular effects of cyclophosphamide, particularly in cancer and immune‐mediated disease therapies [122].

#### 3.1.29. Melatonin

Melatonin is a naturally occurring hormone that is mostly produced by the pineal gland in response to darkness. It plays an important role in sleep–wake cycles and circadian rhythms and several other physiological functions, such as immune regulation, antioxidation, and cell repair. Beyond its well‐established role in sleep regulation, melatonin has garnered attention for its potential therapeutic properties in managing a wide range of health conditions, such as neurodegenerative diseases, cardiovascular disorders, and cancer [123]. Its antioxidant, anti‐inflammatory, and antiapoptotic effects have made melatonin a subject of interest in mitigating tissue damage in conditions like DOX‐induced cardiotoxicity, where it helps reduce oxidative stress and protect myocardial cells from damage [124, 125].

Zhang et al. explored the potential of melatonin in alleviating myocardial damage caused by DOX, with a focus on its effects on mitochondrial function. Although melatonin’s ability to reduce MI through mitochondrial improvement is well‐documented, its role in regulating the sirtuin‐1 (Sirt1)/nuclear factor E2‐related factor 2 (Nrf2) pathway in DOX‐induced cardiomyopathy has not been fully investigated. To address this, the researchers employed H9C2 cardiomyocytes and C57BL/6 mice to develop in vitro and in vivo models of DOX‐induced MI. The results showed that DOX triggered substantial oxidative stress, pyroptosis, and apoptosis, both in vitro and in vivo, but these effects were significantly reduced with melatonin treatment. Mechanistically, melatonin was found to reverse the DOX‐induced downregulation of Sirt1 and Nrf2, and blocking either of these pathways negated the cardioprotective benefits of melatonin. The study concluded that the activation of the Sirt1/Nrf2 pathway is crucial for melatonin’s ability to mitigate oxidative stress, pyroptosis, and apoptosis in DOX‐induced cardiomyopathy, suggesting that melatonin could be a promising therapeutic approach for treating DOX‐induced cardiac injury [126].

#### 3.1.30. Zinc

Zinc is an essential trace element that plays a critical role in numerous physiological processes, including immune function, protein synthesis, wound healing, and DNA synthesis. It is a vital cofactor for over 300 enzymes and is involved in the regulation of cellular growth, division, and apoptosis. Zinc is also known for its antioxidant properties. It has attracted attention for its potential therapeutic applications, particularly in mitigating oxidative stress and inflammation in various diseases, including cardiovascular disorders, diabetes, and cancer [127, 128].

Mohamed et al. examined the protective effects of zinc oxide nanoparticles (ZnO NPs) against MI induced by DOX in rats. The 24 rats were divided into four groups: control, MI, and MI groups treated with two different doses of ZnO NPs (45 mg/kg and 22.5 mg/kg). Treatment with ZnO NPs resulted in significant improvements, including near‐normalization of the ST segment and enhanced cardiac biomarkers such as troponin T, CK, LDH, AST, ALT, alkaline phosphatase, total proteins, MDA, NO, reduced GSH, and CAT. Histological analysis of cardiac and liver tissues showed considerable improvements in the ZnO NPs–treated group. Additionally, the lower dose of ZnO NPs (22.5 mg/kg) proved to be more effective than the higher dose (45 mg/kg). The study concluded that ZnO NPs exhibited strong cardioprotective effects against DOX‐induced MI, likely by enhancing antioxidant defense mechanisms and promoting NO production. This study suggests that ZnO NPs could be a promising therapeutic option for combating DOX‐induced cardiotoxicity [129].

#### 3.1.31. Magnesium

Magnesium is a vital mineral that is involved in different physiological functions, such as muscle activity, nerve functioning, and the control of the cardiovascular system. It is a cofactor for over 300 reactions in the body that are important for energy production, repair of DNA, and protein synthesis. Magnesium also helps maintain normal heart rhythm, supports the immune system, and modulates oxidative stress. Due to its antioxidant properties, magnesium has gained attention in protecting against various forms of stress‐induced damage, including cardiotoxicity. Deficiency in magnesium can lead to a range of health issues, particularly related to cardiovascular function [130].

Khalilzadeh et al. explored whether magnesium sulfate could alleviate cardiac toxicity induced by DOX and whether this effect is associated with modifications in oxidative stress responses in the heart. Male Wistar rats were intraperitoneally injected with DOX, magnesium sulfate, or normal saline four times a week for two consecutive weeks. The combination of magnesium sulfate and DOX significantly reversed the alterations in stimulation threshold and contractile force induced by DOX. Magnesium sulfate also helped preserve body weight and reduce the mortality rate in the treated animals. Histopathological examination showed fewer lesions in the magnesium sulfate‐treated rats. Additionally, magnesium sulfate significantly increased GSH levels in the DOX‐treated animals. The study concludes that magnesium sulfate alleviates DOX‐induced cardiotoxicity by boosting antioxidant enzyme activity [131].

### 3.2. Mitochondrial Bioenergetics

#### 3.2.1. Carnitine

Carnitine is a natural substance that is essential for the movement of long‐chain fatty acids and converts fatty acids into the mitochondria, where they will be oxidized to generate energy. It is synthesized primarily in the liver and kidneys from the amino acids lysine and methionine. Carnitine exists in two forms: L‐carnitine, which is biologically active, and D‐carnitine, which is inactive. It is found in high concentrations in tissues with high energy demands, such as skeletal and cardiac muscles. Beyond its role in fatty acid metabolism, carnitine has been investigated for its potential benefits in various conditions, including cardiovascular diseases, metabolic disorders, and certain forms of cancer therapy‐induced toxicity. It is also available as a dietary supplement and is often used to improve exercise performance and reduce fatigue [132].

Lin et al. examined the relationship between serum carnitine levels and anthracycline‐induced cardiotoxicity in children, adolescents, and young adults (CAYAs). In this prospective pilot study, 21 newly diagnosed patients received a mean cumulative dose of 409 mg/m^2^ DOX equivalents, with cardiac function and serum carnitine measured before, during, and ∼1 year after therapy. LVEF and relative wall thickness declined, accompanied by a reversible drop in serum carnitine. A positive but nonsignificant correlation was observed between carnitine and LVEF (0.09% LVEF decrease per 1 mmol/L‐carnitine reduction, *p* = 0.2). Although the results indicate a possible link between carnitine status and cardiac function after anthracycline therapy, the findings were not statistically significant (*p* > 0.05), and larger studies are required to confirm this relationship [133].

Bayrak et al. in this study aimed to investigate cisplatin (CDDP) induced cardiovascular toxicity and the possible protective effect of acetyl‐L‐carnitine (ALCAR). Three groups with six mice each, one control, one CDDP group (16 mg/kg), and one ALCAR + CDDP group (200 mg/kg ALCAR + 16 mg/kg CDDP) were used in the research.

The study examined several biomarkers, such as caspase‐3, SOD‐2, inducible NOS (iNOS), cyclooxygenase‐2, and Bcl‐2. All of the following parameters (cardiac necrosis, dilated blood vessels, hemorrhage, polymorphonuclear leukocyte infiltration, edema, and pyknotic nuclei) were similar across the groups.

SOD‐2 expression was seen to be increased in the CDDP group, but failed to increase in the ALCAR + CDDP group. There were no significant differences between the levels of iNOS, Bcl‐2, and caspase‐3 in all groups. The results indicate that ALCAR has a protective effect against the cardiotoxic effects of CDDP, and SOD‐2 is an important element in the oxidative stress response that should be investigated as a biomarker of cardiotoxicity [134].

#### 3.2.2. Apigenin

Apigenin may currently exhibit anticancer effects against 14 distinct types of cancer, including bone cancer, glioblastoma, melanoma, lymphoma, leukemia, breast cancer, prostate cancer, colon cancer, bladder cancer, cervical cancer, lung cancer, esophageal cancer, liver cancer, and pancreatic cancer [135]. Apigenin inhibits the growth of leukemia cell lines, induces cell cycle arrest, and causes leukemia cells to undergo apoptosis in vitro [136]. Notably, apigenin does not inhibit the growth of healthy cells, suggesting that it may be a safe anticancer agent for normal tissues. Compared to other structurally similar flavonoids, Hussain et al. also reported that apigenin exhibits lower mutagenic effects and low intrinsic toxicity. According to most of these studies, apigenin primarily induces apoptosis in malignant cells via a mitochondrial‐dependent pathway [137]. Specifically, apigenin downregulates BCL‐2, leading to the release of cytochrome c from the mitochondria into the cytosol and the subsequent activation of caspase‐9 and caspase‐3 [135].

#### 3.2.3. Coenzyme Q10 (CoQ10)

CoQ10 is a naturally occurring lipid‐soluble antioxidant that plays a crucial role in mitochondrial energy production and cellular respiration. It is an essential component of the electron transport chain, facilitating ATP synthesis and protecting cells from oxidative stress. CoQ10 is particularly important for tissues with high energy demands, such as the heart, making it a potential therapeutic agent for cardiovascular diseases, neurodegenerative disorders, and chemotherapy‐induced cardiotoxicity. Studies have shown that CoQ10 supplementation can improve cardiac function, reduce inflammation, and mitigate oxidative damage, highlighting its cardioprotective potential in conditions such as HF and drug‐induced cardiomyopathies [138, 139].

Al‐Hammadi et al. investigated the potential protective effects of CoQ10 against trastuzumab‐induced adverse effects in HER2‐positive breast cancer patients. The study included 100 female patients with HER2+ (HER2+3 or amplified gene) breast cancer, all of whom received standard adjuvant chemotherapy comprising four cycles of ADR, cyclophosphamide, and docetaxel, followed by trastuzumab. The trastuzumab regimen included an initial loading dose of 8 mg/kg, followed by a maintenance dose of 6 mg/kg every 3 weeks for 1 year. The participants were randomly assigned to two groups: One received trastuzumab with a placebo, while the other received trastuzumab along with CoQ10 for a year. The findings revealed that the CoQ10‐treated group showed a significant reduction in levels of monocyte chemoattractant protein‐1 (MCP‐1), IL‐6, soluble Toll‐like receptor 4 (sTLR4), and cTnI compared to the placebo group. However, no significant difference in F2‐isoprostane levels was observed between the two groups. In addition, patients who received CoQ10 showed a significantly smaller decline in ejection fraction (EF) levels at all assessment points except for baseline. These findings indicate that CoQ10 may provide cardioprotective benefits for HER2+ breast cancer patients undergoing treatment with trastuzumab by helping to preserve EF and reduce inflammatory biomarkers and markers of cardiac damage [140].

### 3.3. Modulating Inflammation

#### 3.3.1. Omega‐3

Omega‐3 fatty acids are essential PUFAs that play a crucial role in maintaining cardiovascular, neurological, and immune system health. The three primary types of omega‐3s include ALA, primarily found in plant sources, and the long‐chain fatty acids EPA and DHA, predominantly derived from marine sources such as fish oil. These bioactive lipids exert significant anti‐inflammatory, antioxidant, and cardioprotective effects, making them valuable in the prevention and management of various diseases, including cardiovascular disorders, neurodegenerative diseases, and cancer‐related complications [141].

Monte et al. explored the role of the ceramide pathway in DOX‐induced cardiotoxicity and investigated whether omega‐3 fatty acid supplementation could mitigate its chronic effects in rats. The study included 60 male Wistar rats, which were divided into four groups: Control (C), DOX (D), omega‐3 fatty acids (W), and DOX + omega‐3 fatty acids (DW). Over six weeks, the animals received either omega‐3 fatty acids (400 mg/kg/day, administered via gavage) or water, while DOX (3.5 mg/kg, intraperitoneally) or saline was administered once a week for four weeks. DOX‐treated rats exhibited significant structural and biochemical alterations, including increased left atrial and ventricular diameters, elevated serum triglyceride and cholesterol levels, and heightened oxidative stress markers such as MDA and protein carbonylation. Additionally, these animals showed a reduced left ventricular shortening fraction and a decrease in nSMase1 expression in the heart. Notably, omega‐3 fatty acid supplementation alleviated the structural and functional cardiac damage caused by DOX and reduced protein carbonylation. Furthermore, omega‐3 fatty acids enhanced neutral nSMase activity in both DOX‐treated and untreated rats, although they did not affect nSMase1 protein expression [142].

Similarly, El Amrousy investigated the cardioprotective potential of omega‐3 fatty acids against early DOX‐induced toxicity in children with ALL. This randomized study enrolled 60 newly diagnosed ALL patients, who were assigned to two groups. Group I (*n* = 30) received omega‐3 fatty acids (1000 mg/day) for 6 months alongside their standard chemotherapy regimen, including DOX, whereas Group II (*n* = 30) followed the same chemotherapy protocol without omega‐3 supplementation. After 6 months, Group I exhibited significantly lower MDA levels and higher GSH and SOD levels compared to Group II, suggesting reduced oxidative stress. Additionally, cardiac biomarkers such as troponin I, CK‐MB, and N‐terminal probrain natriuretic peptide (NT‐proBNP) were markedly elevated in Group II but remained unchanged in Group I, highlighting a cardioprotective effect of omega‐3 fatty acids. Echocardiographic assessments further demonstrated that systolic function, measured through peak mitral annular systolic velocity and two‐dimensional global longitudinal strain (GLS), was preserved in omega‐3–treated patients, whereas significant impairment in left ventricular function was observed in the control group. This study was unique in using GLS as a sensitive early marker for detecting subclinical myocardial dysfunction particularly in chemotherapy‐induced cardiomyopathy [143].

Collectively, these studies suggest that omega‐3 fatty acids may serve as a promising intervention to mitigate DOX‐induced cardiotoxicity by reducing oxidative stress and preserving cardiac function, in both preclinical models and pediatric cancer patients.

#### 3.3.2. Hesperidin

Hesperidin is a flavanone glycoside (C_28_H_34_O_15_) found predominantly in the pericarp of citrus fruits. Hesperetin, as its pharmacologically active aglycone form, is generated following intestinal hydrolysis. Beyond its role in plant defense, hesperidin has documented antioxidant, anti‐inflammatory, vasoprotective, antiapoptotic, and antitumor activities across a wide range of experimental and clinical models [144, 145]. Hesperidin has demonstrated cardioprotective efficacy against both DOX and cisplatin through partially distinct but mechanistically convergent pathways. Alharbi et al. demonstrated in Wistar rats that DOX cotreatment with oral hesperidin 100 mg/kg/day for five weeks normalized elevated cardiac biomarkers, including troponin I, CK‐MB, LDH, and AST, suppressed proinflammatory cytokines IFN‐γ, IL‐1β, and TNF‐α, restored SOD, GPx, and CAT activities, and reversed DOX–induced histopathological damage [146]. A particularly novel mechanism has been identified in both acute and tumor‐bearing DOX cardiotoxicity mouse models. Hesperetin sustains CISD2 expression, a mitochondrial protein critical for redox regulation and metabolic homeostasis whose expression is suppressed by DOX, restoring glucose, fatty acid, and amino acid metabolism, reinstating the pentose phosphate and GSH pathways, and improving cardiac electromechanical function without attenuating DOX’s antitumor efficacy [147]. Against cisplatin, hesperidin activates the p62–Keap1–Nrf2 signaling axis by upregulating p62 to competitively inhibit Keap1‐mediated Nrf2 ubiquitination, thereby promoting Nrf2 nuclear translocation and downstream antioxidant gene expression, alongside the reduction of ROS, MDA, TNF‐α, IL‐6, Bax, and caspase‐3, and restoration of SOD, CAT, GSH, and Bcl‐2 [148]. Beyond cardioprotection, hesperidin possesses direct antitumor activity across multiple malignancies by inhibiting proliferation, inducing apoptosis via Bax/Bcl‐2 modulation and caspase activation, suppressing angiogenesis through MMP‐2/MMP‐9 inhibition, and blocking NF‐κB and TGF‐β1/Smad3 signaling [149, 150]. However, a systematic review cautions that hesperidin’s antioxidant and anti‐inflammatory mechanisms may theoretically attenuate the pro‐oxidant cytotoxic mechanisms by which some chemotherapeutics act within tumor cells, underscoring that timing, dosing, and drug‐specific interactions must be rigorously characterized in future clinical trials before hesperidin can be routinely recommended as a cardioprotective adjunct in clinical oncology [151].

#### 3.3.3. Vitamin D

Vitamin D is a vital fat‐soluble nutrient that plays a central role in calcium and phosphate homeostasis, essential for bone health and skeletal function. Beyond its traditional role in bone metabolism, vitamin D has also been implicated in modulating immune responses, reducing inflammation, and protecting against chronic diseases, including cardiovascular disease and certain cancers. The active form of vitamin D, calcitriol, exerts its effects through binding to the vitamin D receptor (VDR) in various tissues, influencing gene expression and cellular functions [152].

El‐Bassiouny et al. investigated vitamin D’s cardioprotective role in 150 women with early breast cancer receiving four cycles of adjuvant DOX–cyclophosphamide chemotherapy (60 mg/m^2^ DOX, 600 mg/m^2^ cyclophosphamide every 21 days). Patients were randomized to chemotherapy alone or plus Bon One (alfacalcidol) 0.5 μg/day, orally throughout treatment. Vitamin D significantly reduced serum LDH, cTnT, and IL‐6 compared to controls, indicating protection against DOX‐induced cardiotoxicity likely via suppression of proinflammatory cytokines [153].

#### 3.3.4. Carnosol

Carnosol is a polyphenolic compound found primarily in rosemary (*Rosmarinus officinalis*) and sage (*Salvia officinalis*), known for its potent antioxidant, anti‐inflammatory, and anticancer properties. It has been shown to possess various health benefits, including neuroprotective, cardioprotective, and hepatoprotective effects, attributed to its ability to modulate oxidative stress and inflammatory pathways. Carnosol exerts its protective effects through mechanisms such as the inhibition of proinflammatory cytokines, modulation of enzyme activities, and activation of cellular defense proteins like sirtuin‐1. Due to its beneficial biological activities, carnosol is increasingly recognized as a potential therapeutic agent for managing conditions associated with oxidative stress, inflammation, and cell damage, such as cardiovascular diseases and cancer [154].

Ghasemzadeh Rahbardar et al. examined carnosic acid’s protective effects against DOX‐induced cardiotoxicity in male Wistar rats. DOX increased QRS duration, heart‐to‐body weight ratio, and oxidative and inflammatory markers, while reducing heart rate, blood pressure, ventricular function, and GSH, causing cardiac fibrosis and necrosis. Carnosic acid (10–40 mg/kg/day) or vitamin E alleviated these changes, lowering MDA, TNF‐α, IL‐1β, caspases, and Bax/Bcl‐2, and improving cardiac structure and function. The authors concluded that carnosic acid’s antioxidant and anti‐inflammatory properties mitigate DOX‐induced cardiac injury, suggesting its potential as an adjunct therapy [155].

Zaabaa et al. investigated carnosol’s protective effects against diesel exhaust particle (DEP)–induced cardiotoxicity in mice. Mice received DEPs (1 mg/kg) or saline intratracheally, with carnosol (20 mg/kg) or saline given intraperitoneally one hour prior. Carnosol significantly lowered DEP‐induced increases in plasma LDH and BNP, normalized proinflammatory cytokines, LPO, and NO and reduced mitochondrial dysfunction, DNA damage, and apoptosis. It also restored sirtuin‐1 expression while suppressing phosphorylated NF‐κB and MAPK activation. The authors concluded that carnosol mitigates DEP‐induced cardiotoxicity via anti‐inflammatory, antioxidant, and antiapoptotic mechanisms, partly through sirtuin‐1 activation and NF‐κB/MAPK inhibition [154].

### 3.4. Emerging Pathways

GPx4 plays a central role in protecting cells against ferroptosis, a form of regulated cell death that depends on iron and is characterized by the buildup of lipid peroxides. GPx4 converts phospholipid hydroperoxides into nontoxic lipid alcohols, using GSH as a cofactor, which helps preserve membrane stability. When GPx4 function is compromised or GSH levels decline, lipid peroxides begin to accumulate. This effect is intensified in the presence of redox‐active iron (Fe^2+^), which drives LPO through Fenton‐type chemistry. As a result, cellular membranes undergo oxidative damage, ultimately leading to ferroptotic cell death. This process is further promoted by elevated ROS, disturbances in iron homeostasis, and weakened antioxidant defenses. In contrast, protective pathways such as Nrf2 signaling can counteract these effects by enhancing the expression of GPx4 and other antioxidant enzymes.

#### 3.4.1. Hydroxytyrosol (HT)

It has been shown that by enhancing the mitochondrial electron transport chain and oxidative damage, HT could successfully reduce DOX‐induced heart damage. The findings imply that HT can interfere with caspase 3 activation and death by blocking the Bax proapoptotic pathway. Its capacity to chelate iron may also contribute to the protective effect of HT in DOX‐mediated oxidative damage. In fact, it has recently been shown that HT, which may chelate iron, can reduce the prolonged phosphorylation of JNK and p38 caused by H_2_O_2_ and labile iron. These result in mitochondrial destabilization, which is followed by the release of cytochrome C, the activation of caspase 3, and cell death [156]. In this regard, DOX’s capacity to stimulate ROS generation and apoptotic activation may be impacted by the decreased iron content. In fact, DOX has a high affinity for iron, and through its interaction with negatively charged membranes, the DOX‐Fe^2+^ complex can stimulate LPO [157]. Recent research has demonstrated that proteins that sequester intracellular iron influence DOX’s action on iron metabolism and that a decrease in DOX via free iron stimulates the redox cycle, which is the cycle of free radical generation. In contrast, because most cells have very low levels of free iron, it is insufficient alone to produce cardiomyopathy under physiological conditions when combined with DOX [7].

#### 3.4.2. Zingerone (ZRN) (From Ginger)

ZRN is a phenolic compound found in ginger (*Zingiber officinale*) that is known for its antioxidant, anti‐inflammatory, and potential cardioprotective properties. It has been shown to modulate oxidative stress, reduce inflammation, and protect against various forms of toxicity, including those induced by chemotherapy drugs. ZRN’s therapeutic potential has attracted attention in several preclinical studies, particularly for its ability to mitigate drug‐induced cardiotoxicity, highlighting its promise as a natural adjunct in protecting against oxidative damage and improving cardiovascular health [158, 159].

Khan et al. conducted a study to assess the cardioprotective effects of ZRN against trastuzumab (TZB)‐induced cardiotoxicity in rats. The experiment involved five groups, each consisting of eight rats. The first group served as the control, receiving normal saline (NC), while the second group received TZB (6 mg/kg/week for 5 weeks) intraperitoneally as the toxic control. Groups 3 and 4 were pretreated with ZRN (50 and 100 mg/kg, orally) alongside TZB for 5 weeks, and Group 5 was treated with ZRN alone (100 mg/kg, orally). TZB administration resulted in cardiotoxicity, indicated by a significant increase in AST, CK‐MB, LDH, and LPO levels. Concurrently, GSH levels and the activity of key antioxidant enzymes, including GPx, glutathione reductase (GR), glutathione‐S‐transferase (GST), CAT, and SOD, were significantly reduced. ZRN pretreatment markedly decreased the levels of AST, CK‐MB, LDH, and LPO while restoring GSH and antioxidant enzyme activities to near normal. Inflammatory cytokines (IL‐2 and TNF‐α) were elevated in the TZB‐only group, but ZRN pretreatment successfully normalized their levels. These findings provide strong evidence for the cardioprotective effects of ZRN against TZB‐induced cardiotoxicity, which was further corroborated by histopathological analysis [158].

Alam et al. evaluated ZRN’s ability to prevent CFZ‐induced cardiotoxicity in Wistar rats. CFZ caused oxidative stress, inflammation, apoptosis, and histopathological heart damage. ZRN (50 or 100 mg/kg) enhanced antioxidant defenses (GSH, CAT, SOD), reduced IL‐1β, IL‐6, TNF‐α, and caspase‐3 levels, and improved cardiac histology. The study concluded that ZRN protects against CFZ‐induced cardiac injury through antioxidant, anti‐inflammatory, and antiapoptotic effects [159].

#### 3.4.3. Lycopene (LYC)

LYC is a naturally occurring carotenoid pigment found in various fruits and vegetables, with tomatoes being the most notable source. It is a powerful antioxidant, recognized for its ability to neutralize free radicals and reduce oxidative stress. LYC has been linked to several health benefits, including its potential role in lowering the risk of cardiovascular diseases, certain cancers, and age‐related conditions. Recent studies have investigated its therapeutic potential in alleviating the toxic effects of chemotherapy agents, underscoring its promise as a natural supplement for improving health outcomes across various disease models [160, 161].

Huang et al. investigated the protective effects of LYC against DOX‐induced HF and explored the underlying mechanisms. The study utilized DOX to establish an HF model in cardiomyocytes and C57BL/6J mice, focusing on how LYC impacted inflammation, oxidative stress, and ferroptosis in the context of DOX‐induced HF. The results showed that LYC effectively alleviated DOX‐induced cardiac dysfunction. Mechanistically, LYC reduced collagen deposition and fibrosis by inhibiting the expression of matrix metalloproteinases MMP‐2 and MMP‐9. Additionally, LYC suppressed the production of ROS by enhancing antioxidant enzyme activity. It also increased B‐cell lymphoma 2 (Bcl‐2) expression, reducing apoptosis by lowering the levels of tumor protein 53 (p53), Bcl‐2‐associated X protein (Bax), and cleaved caspase‐3 (c‐Casp3). Moreover, LYC reduced inflammatory cytokine levels through the activation of peroxisome proliferator‐activated receptor gamma (PPARγ). The study also demonstrated that LYC attenuated DOX‐induced ferroptosis, both in vivo and in vitro, by activating Nrf2. These findings suggest that LYC mitigates DOX‐induced cardiac dysfunction by targeting oxidative stress, inflammation, and ferroptosis, particularly through Nrf2 pathway activation, highlighting its potential as a therapeutic agent for HF [162].

Lin et al. investigated the effects of ATR and/or LYC on cardiac health by administering ATR (50 mg/kg or 200 mg/kg) and/or LYC (5 mg/kg) to mice via intragastric administration for 21 days. The study revealed that ATR decreased CK activity and caused significant histological damage. ATR exposure also altered ion concentrations (Na+, K+, and Ca2+) and led to the reduced activity of Na+‐K+‐ATPase and Ca2+‐ATPase, as well as decreased mRNA expression of their subunits. Notably, supplementation with LYC provided significant protection against ATR‐induced heart damage. In conclusion, ATR induced cardiotoxicity by modulating cardiac ATPase activity and subunit transcription, resulting in ionic disturbances. However, LYC supplementation effectively counteracted these effects by regulating ATPase activity and subunit transcription, demonstrating its promising potential as a chemopreventive agent against ATR‐induced cardiotoxicity [163].

#### 3.4.4. Fisetin

A bioactive polyphenolic flavonoid, fisetin (3, 7, 39, 49‐tetrahydroxyflavone), is present in a wide variety of fruits and vegetables, including cucumbers, apples, strawberries, persimmons, and onions [164]. Fisetin has antioxidant, anti‐inflammatory, antiapoptotic, anticancer, hypolipidemic, neuroprotective, and senolytic activities. [165, 166]. The main mechanism responsible for fisetin’s cardioprotection against DOX involves the inhibition of ferroptosis, an iron‐dependent, ROS‐mediated form of cell death increasingly recognized as a key driver of anthracycline cardiomyopathy. Fisetin activates the Sirt1/Nrf2 signaling axis, upregulating GPX4 and GSH, thereby reversing DOX‐induced LPO, myocardial fibrosis, and cardiac hypertrophy [167]. Beyond ferroptosis, fisetin represses the classical apoptotic and inflammatory cascades: It significantly reduces caspase‐3, COX‐II, iNOS, TNF‐α, and IL‐1β expression in addition to restoring SOD, GSH, and MDA to near‐normal levels and normalizes ECG, hemodynamic, and left ventricular functional parameters in DOX‐treated rats [168]. Lin et al. proposed that fisetin decreases cardiotoxicity by estrogen receptor‐α/β activation, which leads to the inhibition of the IGF‐IIR apoptotic pathway [169]. Crucially, in tumor‐bearing models, the combination of fisetin with DOX demonstrated synergistic antitumor activity in lymphoma, indicating that fisetin’s cardioprotection does not compromise, and may enhance, anticancer efficacy [170]. This synergistic effect has also been shown with paclitaxel and cisplatin in lung cancer models [171, 172].

## 4. Safety and Drug Interactions of Cardioprotective Agents in DOX Therapy

While numerous cardioprotective compounds—including antioxidants, flavonoids, amino acids, and natural products—have demonstrated efficacy in mitigating DOX‐induced cardiotoxicity, their concurrent use with chemotherapy requires careful consideration due to potential antioxidant paradoxes and pharmacokinetic interactions.

## 5. The Antioxidant Paradox and Therapeutic Window

The “antioxidant paradox” arises from the dual role of ROS in both cardiotoxicity and tumor killing. Anthracyclines depend partly on ROS‐mediated DNA damage and topoisomerase II inhibition for antitumor efficacy, raising concern that potent, broad‐spectrum antioxidants such as NAC and vitamin E might protect cancer cells when given concurrently with chemotherapy [173, 174]

Vincent et al. note that early clinical and experimental data on anthracyclines plus antioxidants show mixed effects on tumor response, and although ROS may not be the sole driver of cell death, ROS‐dependent cytotoxicity likely contributes to efficacy [175].

Available human trials of NAC, vitamin E, CoQ10, and related agents are limited by small samples and heterogeneous timing and dosing, underscoring the need for prospective, randomized studies that stratify by administration schedule. Until larger, high‐quality evidence emerges, systemic broad‐spectrum antioxidants should be used cautiously with anthracycline‐based regimens, and preference should be given to agents with more selective mechanisms (e.g., iron chelation, mitochondrial‐targeted or anti‐inflammatory effects) that may preserve antitumor ROS signaling while reducing cardiotoxicity [176].

## 6. Pharmacokinetic Considerations

Several natural compounds commonly used for cardioprotection can influence drug metabolism, particularly through the inhibition of cytochrome P450 enzymes (e.g., CYP3A4, CYP2C9, CYP2D6) and drug transporters such as P‐glycoprotein (P‐gp):

• **Curcumin** inhibits CYP3A4, CYP2C9, and P‐gp, potentially slowing DOX clearance.
• **Black seed oil (TQ)** inhibits CYP3A4, which may increase systemic exposure to chemotherapeutic agents.
• **Resveratrol and pterostilbene** also exhibit CYP3A4 inhibitory activity, warranting caution in polypharmacy settings.
• **Quercetin** inhibits CYP3A4, CYP2C9, CYP2D6, and P‐gp potentially slowing DOX clearance.
• **Genistein, luteolin, apigenin, fisetin, baicalein, silymarin, propolis, and capsaicin** inhibit CYP3A4 moderately and may inhibit P‐gp, warranting caution while using with chemotherapeutic agents.

These interactions highlight the necessity to evaluate potential drug–drug interactions when considering adjunctive use of natural cardioprotective compounds.

## 7. Conclusion

Chemotherapy‐induced cardiotoxicity limits curative cancer care through oxidative stress, mitochondrial injury, inflammation, and regulated cell death. Evidence suggests certain nutraceuticals may aid cardioprotection, with the strongest signals for omega‐3 fatty acids, alpha‐lipoic acid in anthracycline and taxane regimens, and early promise for vitamin D, vitamin E, and levocarnitine.

A summary of available supporting data has been highlighted below. Levels of evidence (LOE) and classes of recommendation (COR) were determined and are presented in Tables 1 and 2 [177]. Evidence levels of nutraceuticals with possible protective effects for chemotherapy‐induced cardiotoxicity are reported in Tables 3 (clinical) and 4 (animal studies).

**TABLE 1:** Revised (2024) ESC level of evidence.

Level	Description
Level A	Data derived from multiple randomized clinical trials or their meta‐analysis
Level B1	Suggestive evidence from ≥ 1 adequately powered RCT or meta‐analysis.
Level B2	Limited evidence from nonrandomized studies or small RCTs.
Level C	Preliminary evidence or expert consensus.

**TABLE 2:** ESC classes of recommendation.

Classes of recommendations	Definition
Class I	Evidence and/or general agreement that a given treatment or procedure is beneficial, useful, and effective
Class II	Conflicting evidence and/or a divergence of opinion about the usefulness/efficacy of the given treatment or procedure
Class IIa	The weight of evidence/opinion is in favor of usefulness/efficacy
Class IIb	Usefulness/efficacy is less well established by evidence/opinion (usefulness and effectiveness is unclear/may be considered)
Class III	Evidence or general agreement that the given treatment or procedure is not useful/effective and, in some cases, may be harmful

**TABLE 3:** Clinically supported nutraceuticals for combating chemotherapy‐induced cardiotoxicity: evidence from human trials and observational studies.

Nutraceutical compound (study name)	Classes (strength) of recommendations	Levels of evidence	Subjects (population)	Sample size	Duration of studies	Consumed dose	Effects on chemotherapy‐induced cardiotoxicity	Side effects and toxicity
N‐Acetylcysteine [15]	Class IIa	Level B2 (single RCT)	Cancer patients receiving chemotherapy	*N* = 60	2 months	600 mg/day oral	Reduced mean level of Troponin I (*p* < 0.001)	Mild, no serious toxicity

Carnitine [133]	Class IIb	Level B2 (cohort study)	Observational cohort patients	*N* = 21	12 months	Carnitine levels were measured	Declined LVEF and relative wall thickness, a reversible drop in serum carnitine	No significant adverse events
							0.09% LVEF decrease per 1 mmol/L‐carnitine reduction (*p* = 0.2)	

Alpha‐lipoic acid [30]	Class IIa	Level B1 (single RCT)	Women with breast cancer (doxorubicin + cyclophosphamide followed by paclitaxel)	*N* = 64	6 months	600 mg/day	reduction in biomarkers like BNP, TNF‐α, MDA, and nitrotyrosine (NT) suggested cardioprotection (*p* < 0.05)	No significant adverse events

Omega‐3 fatty acids [143]	Class IIa	Level B2 (single RCT)	Children with acute lymphoblastic leukemia (ALL)	*N* = 60	6 months	1000 mg/day omega‐3	Preserved left ventricular function ↓oxidative stress, cardiac biomarkers: troponin I, CK‐MB, NT‐proBNP (*p* value not mentioned)	Not reported

Black seed oil [114]	Class IIa	Level B2 (single RCT)	Children with acute lymphoblastic leukemia	*N* = 40	One week	80 mg/kg/dose oral	Preserved systolic function (EF, FS, s wave) with intervention (*p* value not mentioned)	No serious adverse events reported

Vitamin E [90]	Class IIa	Level B1 (single RCT)	Doxorubicin‐treated patients	*N* = 74	4 chemo cycles (months)	Not mentioned	significantly lower levels of BNP (*p* < 0.001) and creatine kinase	No serious adverse events
							(*p* < 0.001) reduction in cardiac events by 24.2% (*p* = 0.02)	

Vitamin D [153]	Class IIa	Level B1 (single RCT)	Doxorubicin‐treated patients	*N* = 150	4 chemo cycles (3 months)	0.5 μg/day, oral	reduced serum LDH, cTnT, and IL‐6 (*p* < 0.001)	No major side effects

Coenzyme Q10[140]	Class IIa	Level B1 (Single RCT)	HER2+ breast cancer patients	*N* = 100	12 months	200 mg oral	Decrease in the reduction in EF levels after 3, 6, 9, and 12 months (*p* = 0.001, *p* = 0.043, *p* = 0.001, *p* = 0.001)	Mild gastrointestinal discomfort
							↓ Cardiac injury biomarkers MCP‐1, IL‐6, sTLR4, and cTnI (*p* < 0.05)	

**TABLE 4:** Nutraceuticals with preclinical evidence for mitigating chemotherapy‐induced cardiotoxicity: Insights from animal model studies.

Nutraceutical compound (study name)	Species	Tumor‐bearing status	Human equivalent dose (HED)	Description of study findings (abstracted)
Taurine [24]	Systematic review of nonclinical studies	N/A	500–3000 mg/day^∗∗^ [178]	‐ Attenuates oxidative stress, inflammation, and apoptosis
				‐ Preserves cardiac structure and function in chemotherapy models

Rutin			3.24–6.49 mg/kg^∗∗∗^	
[38]	Wistar Albino male rats (20–40 mg/kg)	Healthy		‐ Attenuates carfilzomib‐induced cardiotoxicity by inhibiting NF‐κB
[36]	Rats	Healthy		‐ Lessens cardiac enzyme alterations (LDH, CK)
[37]	Rats	Healthy		‐ Protects against tacrolimus‐induced cardiotoxicity

Naringenin [44]	Male Wistar rats (50 mg/kg/day)	Healthy	8.1 mg/kg/d^∗∗∗^	‐ Decreases CTnT, LDH, CK‐MB
				‐ Lowers IL‐6, CRP, NO, ACE
				‐ Reverses ECG abnormalities

Acetyl‐L‐carnitine [134]	Mice	Healthy	500–2000 mg/day^∗∗^ [179]	‐ Provides biochemical cardioprotection
				‐ Antioxidant effects without significant structural changes

Betaine [135]	Rats	Healthy	∼1.5 g–3 g/day^∗∗^ [17]	‐ Reduces oxidative stress, inflammation, and fibrosis
				‐ Modulates AMPK/Nrf2/TGF‐β signaling pathways

Carnosine [20]	Rats	Healthy	Up to ∼10 g/day^∗∗^ [180]	‐ Alleviates cardiac toxicity and histopathological damage
				‐ Enhances antioxidant effects with vitamin E

Glutamine [22]	Rats	Mammary adenocarcinoma	∼5 g–30 g/day^∗∗^ [181]	‐ Decreases cardiotoxicity biomarkers
				‐ Does not compromise anticancer efficacy

Baicalein [33]	Male Swiss Albino Mice (100 mg/kg/d, orally) for 4 wk	Healthy	8.11 mg/kg^∗∗∗^	‐ Decreases cardiac biomarkers and reverses GSH reduction

Genistein [66, 163]	Mice	Healthy	Not Reported	‐ Decreases lipid peroxidation and ROS production

Caffeic acid [75]	Rats 40 mg/day	Healthy	assume 0.25 kg rat ⟶ HED = ∼6.49 mg/kg^∗∗∗^	‐ Improves LV function, reduces cytokine expression and myocardial injury (↓cTn‐I)

Silymarin [52]	Male Wistar rats	Healthy	9.73 mg/kg^∗∗∗^	‐ Protects against DOX toxicity

Pterostilbene [65]	C57BL/6 mice (10 mg/kg/day IP)	Healthy	0.81 mg/kg^∗∗∗^	‐ Protects cardiomyocytes from oxidative stress and mitochondrial damage

Curcumin			∼500–2000 mg/day (standardized)^∗∗^ [182]	‐ Reduces early electrocardiographic signs of cardiotoxicity, antioxidant enzymes, and cardiac tissue degeneration ‐ Lowers cardiotoxicity caused by chemotherapeutic agents
[68]	Albino rats	Healthy		
[69]	Rats	Healthy		
[71]	Rats	Healthy		
[72]	Rats	Healthy		

Vitamin C [88]	Rats	Healthy	90 mg/day (men), 75 mg/day (women); UL 2000 mg/day^∗^	‐ Preserves cardiac function and prevents structural myocardial damage

Zingerone [158]	Rats 50 and 100 mg/kg	Healthy	8.11–16.22 mg/kg^∗∗∗^	‐ Lowers oxidative damage, inflammation, and apoptosis
				‐ Protects against chemotherapy‐induced cardiac toxicity

Carnosol [154, 155]	Male Wistar rats (carnosic acid (40 mg/kg/day IP)	Healthy	6.49 mg/kg^∗∗∗^	‐ Inhibits inflammation, oxidative stress, apoptosis, and fibrosis
	Mice	Healthy		‐ Improves cardiac function and structure

Cyanidin 3‐glucoside [97]	Mice	Healthy	Not reported	‐ Improves survival of cardiomyocytes and reduce histopathological damage

Rosmarinic acid [98]	Male rats (40 mg/kg, IP)	Healthy	6.49 mg/kg^∗∗∗^	‐ Ameliorates toxic effects of DOX

Lycopene [162, 163]	Mice	Healthy	0.41 mg/kg^∗∗∗^	‐ Attenuates fibrosis, apoptosis, oxidative stress, and inflammation
	Mice (5 mg/kg)	Healthy		‐ Enhances cardiac function and survival

Fisetin [168]	Male Sprague‐Dawley rats (20–40 mg/kg/day)	Healthy	3.2–6.5 mg/kg/day^∗∗∗^	‐ Decreases CK‐MB, LDH, cTn‐I, caspase‐3, TNF‐α, IL‐1β
				‐ Normalizes ECG and LV function
				‐ Increases SOD, GSH; lowers MDA

Capsaicin [122]	Rats	Healthy	∼2–10 mg/day^∗∗^ [183]	‐ Mitigates oxidative stress and inflammation
				‐ Restores antioxidant enzymes and cardiac enzyme levels

Melatonin [126]	Rats	Healthy	∼0.5–5 mg bedtime^∗∗^ [184]	‐ Activates Sirt1/Nrf2 pathway
				‐ Reduces oxidative stress, pyroptosis, and apoptosis

Selenium [94]	Male rats	Healthy	55 μg/day; UL 400 μg/day^∗^	‐ Enhances antioxidant enzyme activity
				‐ Lowers cardiac injury markers and histopathological changes

Zinc [129]	Rats	Healthy	11 mg/day (men), 8 mg/day (women); UL 40 mg/day^∗^	‐ Improves cardiac biomarkers, antioxidant status, and histological cardiac and liver tissue appearance—enhances antioxidant defense

Magnesium [131]	Male Wistar rats	Healthy	420 mg/day (men), 310 mg/day (women); UL (supplement) 350 mg/day^∗^	‐ Prevents cardiac lesions and mortality
				‐ Boosts glutathione levels and preserves cardiac function

*Note:* Km factors (FDA standard): mouse = 3 rat = 6 human (60 kg) = 37.

Abbreviation: IP = intraperitoneal.

^∗^RDA [185].

^∗∗^Typical safe dose (humans).

^∗∗∗^HED (mg/kg) = animal dose (mg/kg) × [Km (animal)/Km (human)] [186].

Preventive cardio‐oncology strategies should pair risk stratification with sensitive biomarkers such as cardiac troponin, natriuretic peptides, and advanced echocardiography to detect subclinical injury and guide timely intervention. Adoption is limited by heterogeneous study designs, uncertain bioavailability, product quality, and drug–nutrient interactions. Progress requires large, standardized multicenter trials with defined endpoints, quality control, and integration of pharmacokinetics and nutrigenomics. Nutraceuticals should complement, not replace, established cardioprotective measures, within coordinated oncology‐cardiology‐nutrition care.

## Author Contributions

Seyedeh‐Tarlan Mirzohreh, Samad Ghaffari, and Neda Roshanravan conceptualized and designed the study. Literature review was performed by Seyedeh‐Tarlan Mirzohreh, Naimeh Mesri Alamdari, Erfan Banisefid, Melika Ghaffari, Arvin Namazi Shabestari, Armin Imanzadeh Ghazijahani, and Faezeh Tarighat. Seyedeh‐Tarlan Mirzohreh and Neda Roshanravan prepared the first draft of the manuscript. All authors contributed to the critical revision of the manuscript for intellectual content.

## Funding

The Research Vice Chancellor of the Tabriz University of Medical Sciences sponsored the study (Grant No. 79182).

## Disclosure

All authors approved the final version.

## Ethics Statement

The study protocol was approved by the Ethics Committee of Tabriz University of Medical Sciences (Ethics Code: IR.TBZMED.REC.1405.122).

## Consent

This article is a narrative review based on the previously published literature. No consent was required.

## Conflicts of Interest

The authors declare no conflicts of interest.

## Data Availability

No new data were generated or analyzed in this study. Data sharing does not apply to this article.
